# Phage Therapy, a Salvage Treatment for Multidrug-Resistant Bacteria Causing Infective Endocarditis

**DOI:** 10.3390/biomedicines11102860

**Published:** 2023-10-22

**Authors:** Helal F. Hetta, Zainab I. Rashed, Yasmin N. Ramadan, Israa M. S. Al-Kadmy, Soheir M. Kassem, Hesham S. Ata, Wedad M. Nageeb

**Affiliations:** 1 Department of Medical Microbiology and Immunology, Faculty of Medicine, Assiut University, Assiut 71515, Egypt; helalhetta@aun.edu.eg; 2 Department of Microbiology and Immunology, Faculty of Pharmacy, Assiut University, Assiut 71515, Egypt; zeinab_rashed@pharm.aun.edu.eg (Z.I.R.); yasmine_mohamed@pharm.aun.edu.eg (Y.N.R.); 3 Branch of Biotechnology, Department of Biology, College of Science, Mustansiriyah University, Baghdad P.O. Box 10244, Iraq; 4 Department of Internal Medicine and Critical Care, Faculty of Medicine, Assuit University, Assiut 71515, Egypt; soheir@aun.edu.eg; 5 Department of Pathology, College of Medicine, Qassim University, Buraydah 51452, Qassim, Saudi Arabia; hsad@qu.edu.sa; 6 Department of Medical Microbiology and Immunology, Faculty of Medicine, Suez Canal University, Ismailia 41522, Egypt; wedad_saleh@med.suez.edu.eg

**Keywords:** phage, phage therapy, infective endocarditis (IE), MDR, IE management

## Abstract

Infective endocarditis (IE) is defined as an infection of the endocardium, or inner surface of the heart, most frequently affecting the heart valves or implanted cardiac devices. Despite its rarity, it has a high rate of morbidity and mortality. IE generally occurs when bacteria, fungi, or other germs from another part of the body, such as the mouth, spread through the bloodstream and attach to damaged areas in the heart. The epidemiology of IE has changed as a consequence of aging and the usage of implantable cardiac devices and heart valves. The right therapeutic routes must be assessed to lower complication and fatality rates, so this requires early clinical suspicion and a fast diagnosis. It is urgently necessary to create new and efficient medicines to combat multidrug-resistant bacterial (MDR) infections because of the increasing threat of antibiotic resistance on a worldwide scale. MDR bacteria that cause IE can be treated using phages rather than antibiotics to combat MDR bacterial strains. This review will illustrate how phage therapy began and how it is considered a powerful potential candidate for the treatment of MDR bacteria that cause IE. Furthermore, it gives a brief about all reported clinical trials that demonstrated the promising effect of phage therapy in combating resistant bacterial strains that cause IE and how it will become a hope in future medicine.

## 1. Introduction

Infective endocarditis (IE) is an infection of the endocardium, or inside surface of the heart, which most usually affects the heart valves or implanted cardiac devices [1]. IE affects both the left and right sides of the heart. Most of the time, people with left-sided IE are thought to have more severe and complicated infections than those with right-sided IE. According to reliable reports, IE is still relatively uncommon but is increasing in prevalence, mostly in high-income countries [1,2].

IE is a potentially fatal heart infection that is more likely to arise in some persons who have numerous cardiac valve disorders [3]. Its yearly incidence is between 3 and 10 per 100,000 people [4]. IE is still one of the most fatal diseases in the world, with an overall fatality rate of about 25% [4,5].

According to a demographic survey conducted in France, the age- and sex-standardized yearly incidence of IE was 33.8 cases per million people, with a 22.7% overall in-hospital death rate [6]. Similarly, significant IE death rates have been observed in various poor nations [7]. Additionally, the cost of IE hospitalizations in the United States increased dramatically over time, going from USD 1.56 billion in 2003 to USD 2.34 billion in 2016 [8].

The diagnosis and management of IE have advanced quickly yet vary widely in various areas and nations, leading to the same significant global variations in disease burden. Rheumatic heart disease, the use of prosthetic valves or cardiac devices, the use of intravenous drugs, and other blood-borne bacterial infections are only a few of the risk factors that contribute to IE morbidity [9]. In addition, there are varying levels of response capacity across different areas due to the complicated and variable clinical manifestations and courses of IE [10].

Endocarditis can be diagnosed with the modified Duke criteria. These criteria have an average sensitivity of 80%; however, this percentage drops dramatically in situations involving implanted electronic device infections or prosthetic valve endocarditis. Clinical suspicion, microbiological correlation, and further imaging with whole-body computed tomography (CT), cerebral magnetic resonance imaging (MRI), or increasingly, 18F-labeled fluoro-2-deoxyglucose positron emission tomography (18F-FDG-PET)/CT, may be necessary for this situation [11]. A specialized endocarditis team at a reference center must deal with complicated scenarios of endocarditis that are preceded by structural damage (development of an abscess, a perforation, or a fistula), heart failure, and valve incompetence [12]. Latent cases may be go ignored in communities that have not kept up with updated diagnostic techniques, such as 18F-FDG PET scans for patients with implanted devices and valves, which have a better diagnostic sensitivity [13]. Drug resistance (DR) is an additional challenge.

Multidrug-resistant (MDR), extensively drug-resistant (XDR), and pan-drug-resistant (PDR) phenotypes of resistant bacteria have all recently been identified. Accordingly, MDR and XDR bacteria pose a severe danger to public health and the effectiveness of frequently prescribed antibiotics [14,15,16,17,18,19,20,21]. MDR, XDR, and PDR Gram-negative bacteria have been steadily increasing in recent years, and most cases have been reported to be affected by MDR or XDR *Mycobacterium tuberculosis*, *Acinetobacter baumannii*, *Escherichia coli*, *Klebsiella pneumoniae*, and *Pseudomonas aeruginosa* [22,23,24,25,26,27,28,29,30,31,32,33,34,35,36]. Antibiotic resistance has been spread between bacteria through different mechanisms, including the intrinsic genetic and physiological transmission that occurs vertically across species as well as the propensity of bacteria to exchange various genes horizontally between species and genera [37,38,39,40,41,42,43,44].

The hazards of infections caused by complicated pathogens are not effectively tackled by the alternative therapies currently available, thus researchers are urged to develop novel solutions to the growing problem of bacterial MDR, XDR, and PDR. Even though a range of management methods have been employed to combat resistant pathogens, such as (a) understanding of the molecular origins, evolutionary history, and spread of resistance; (b) identifying novel chemical substances with antibacterial activity; and (c) using combinatorial therapy to increase the efficacy of antibiotics. Despite these management methods, the number of resistant microorganisms is increasing. So, other strategies are needed to control the spread of resistant bacteria. Phage therapy is considered one of these strategies [45,46,47]. Bacteriophages are certain viruses that naturally infect bacteria and have been employed as an antibacterial treatment. Phage therapy is employed by using phages as antibacterial substances that can cause lysis of targeted pathogenic microorganisms [48].

Given the background of rising antibiotic resistance and dwindling new antibacterial drugs, phage therapy, a revolutionary safe technique, is attractive for the following reasons: phages are easily isolated from a variety of surroundings, and only affect the infected cell, without affecting neighboring cells [48]; they successfully overcome MDR, XDR, and PDR bacteria; and they may be able to take the place of antibiotic therapies [49,50,51,52].

Due to the concerning growth in antibiotic resistance among bacteria, bacteriophages have attracted interest as potential antimicrobial entities in the Western scientific community. Although bacteriophages are typically thought of as prokaryote-specific viruses, current research shows that they can interact with eukaryotic species, including humans [53]. Within a eukaryotic cell, phages can penetrate and spread transcytosis, which involves the endomembrane systems of eukaryotic cells, particularly the Golgi apparatus, which is necessary for bacteriophage cell penetration [54]. The beginning of transcytosis occurs when a bacteriophage particle is taken up by the cell membrane and moved into the cytoplasm inside a tiny vesicle. The vesicle subsequently passes through the Golgi apparatus and is released on the opposite side of the same cell. The procedure was repeated by the adjacent cells, allowing the bacteriophage particle to cross cell layers [54].

A search for workable substitutes has been sparked by the increased incidence of MDR bacteria and the inadequate effectiveness of current antibiotics in treating infectious diseases. Bacteriophages and the endolysins that are connected with them may be effective weapons against MDR pathogens [55,56,57].

## 2. Bacteriophages: What Are They?

Bacteriophages are viruses that attack and infect bacteria. They have every property that viruses have in common. Being small (50–200 nm) and carrying the genetic instructions for quick and effective replication, phages cannot replicate on their own and need a bacterial host to do so. Like other viruses, they are normally unique to a certain bacterial host; any one phage may infect many different species within a genus and most or many strains within a species, but occasionally just one or very few isolated individuals of a species. Only a small percentage of phages can infect bacteria from various genera, and they normally accomplish this when they are phylogenetically related. The range of bacteria that a particular phage chooses to infect is known as the host range, and it can be extremely limited (just a few bacterial isolates can support its reproduction) or extremely vast (infecting several distinct species or even different genera). Host range, or host “preference”, is a crucial element affecting the therapeutic potential of phages [58].

Phages are ubiquitous and numerous in the environment; ocean water has 107 phage particles per mL [59], and the number of phage particles in the biosphere is believed to be ten times greater than the number of bacteria [60,61].

There is a great deal of genetic variability among phages, and it is uncommon to separate two individuals with the same genetic makeup, though the degree of variability differs depending on the host [62].

By using electron microscopy, which shows a wide range of amazingly different shapes and sizes, phages can be easily seen. However, the order Caudovirales, which has tail-borne double-stranded DNA (dsDNA)-carrying phages, makes up most phages present in the surroundings. The dsDNA is located in the head of each of these phages, while the receptor recognition activities are found at the tip of the tail. According to the types of tails they have, phages can often be grouped into three morphotypes: siphoviridae (long, noncontractile tails), myoviridae (contractile tails), and podoviridae (short, stubby tails) [58].

**Figure 1:**
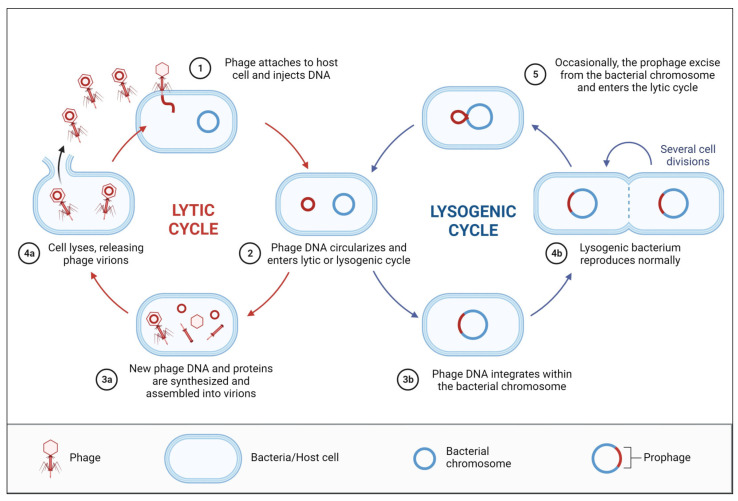
Life cycles of bacteriophages. (a) The primary mechanisms by which lytic phages infect their host bacteria are adsorption, DNA injection, DNA replication, and bacterial cell lysis, The only outcomes are phage growth and cell death of the lytic cycle, but (b) temperate phages “choose” between lytic growth and lysogeny, in which the phage genome is integrated into the host chromosome (creating a prophage), and the lytic genes are turned off, when they attach to their host bacteria and inject their DNA. So, the biological basis of phage therapy is the death of phage-infected bacterium. Created with BioRender.com.

Phages can also be divided into two groups: virulent and temperate (Figure 1). This distinction is crucial when thinking about potential therapeutic applications. For virulent phages, there is only one rational response to infection: phage replication, lysis of the bacterial cell, and the release of phage progeny. Although the lytic cycle is a potential scenario of infection for temperate phages, another potential scenario is the lysogenic cycle. In the lysogenic cycle, prophage DNA is stably preserved within the bacterium (often by integration into the host genome), the genes required for lytic development are turned off, and the lysogenic cell continues to grow and divide. The ecosystem is populated with both varieties of phages [63].

## 3. History of Phage Therapy

Two microbiologists, Felix d’Herelle in 1917 and Frederik Twort in 1915, independently discovered phages. However, Twort was the first to notice and explain the results of a “transparent substance” that slowed down bacterial growth [64]. The term “bacteriophage”, often known as a “bacteria-eater,” was not first used to describe an obligate bacterial parasite until 1917, when d’Herelle identified an anti-*Shigella* microbe [65]. D’Herelle recognized the therapeutic potential of phage as a cure for bacterial disorders. He successfully treated hens infected with *Salmonella gallinarum* in 1919 using phages [66,67]. Following his success in treating animal diseases, d’Herelle soon tried using phages to treat human infections. In 1921, five patients with bacillary dysentery had successful outcomes when treated with a phage that infects *Shigella dysenteriae* [66,68]. In 1927, a clinical study for cholera therapy in India indicated that the mortality rate dropped from 62.8% in control groups to 8.1% in phage-treated groups [69]. Moreover, d’Herelle stated that infusing anti-cholera phage into drinking well water during an outbreak slowed the spread of new infections [69].

## 4. Early Recognized Obstacles for Phage Therapy

Following d’Herelle’s initial results, other researchers, with various degrees of success, began to focus on other infections after realizing the treatment and preventative possibilities of phage. Scientists began noticing several potential difficulties with phage therapy regarding the design and quality of early phage therapy experiments: (1) The potential drawback of high phage specificity was discovered, revealing that phages may be ineffective in the lack of bacterial sensitivity knowledge [56]. (2) Early techniques for synthesizing therapeutic phages in large quantities were probably highly contaminated with lysed bacteria. It was challenging to distinguish between the potential benefits of phages and the confusing effects of contaminating bacterial antigens due to the limited and inconsistent filtering and purification stages [70]. (3) Early pharmacokinetic studies revealed that phages were quickly eliminated from the body through the spleen, raising doubts about their long-term usefulness [71]. (4) Bacteria are easily capable of developing phage resistance, as reported by Luria and Delbruck. They used selection by lytic phage in 1943 to determine the rates of spontaneous mutation in bacteria [72]. (5) Lastly, early research revealed that experimental results shown in vivo did not necessarily match with those seen in vitro [73,74]. Due to the above-mentioned problems and the availability of newly developed antibiotics, interest in phage therapy decreased, starting during the 1970s, the Western world began to move away from phage therapy [56].

## 5. Innovative Studies by Smith and Huggins

Smith and Huggins started by proving that the in vitro and in vivo effectiveness of phages may be associated; also, they also further characterized phage R, which exhibited the greatest in vitro pathogenicity [75]. Phage R appears to have a limited host range and most likely uses the K1 capsule as a receptor to infect exclusively K1+ *E. coli*. Using a series of lethal bacterial challenges in mice, Smith and Huggins demonstrated that a single dose of phage R was comparable to eight doses of streptomycin [75]. Moreover, Smith and Huggins looked at the stability of phage during oral therapy in 1987 and found that giving calcium carbonate before phage could help with poor phage stability in the stomach’s acidic environment [76].

Then, Smith and Huggins’ results were updated by other researchers. Although rapid phage clearance in vivo was formerly thought to be a drawback of phage therapy, Merril et al. showed that it is possible to choose phage types that have long blood circulation [77].

Soothill proved the effectiveness of phage therapy in mice infected with either *P. aeruginosa* or *A. baumanii* following Smith and Huggins’ successful outcomes in treating *E. coli*-infected animals with phages [78]. In 2002, Bull and Levin et al. replicated Smith and Huggins’ initial findings when comparing the efficiency of a K1-antigen-targeting phage with a non-K1-targeting phage against *E. coli* in mice [79]. In contrast to the non-K1 targeting phage, which caused 60% mortality in treated mice, the K1-targeting phage was seen to protect 100% of the animals. Phage treatment caused 9% fewer deaths in a subsequent experiment than streptomycin, which caused 54% more deaths. These findings support those of Smith and Huggins, who found that K1-targeting phages are more successful in treating disease than non-K1-targeting phages or antibiotics [79].

## 6. Phage Therapy: An Updated Strategy

The definition and testing of phages as an antibacterial medicine have greatly improved. Modern technology makes it possible to examine hundreds or even thousands of samples at once using efficient high-throughput methods, low-cost whole-genome sequencing, and automated microbial growth monitoring. In comparison to earlier attempts, advanced clinical trials should be precisely designed to be safer and more inclusive, as well as yield relevant data. The double-blinded, placebo-controlled, and diversified cohort are characteristics of an appropriate phage therapy trial. Using clinical isolates may also be planned to generate useful longitudinal data. For instance, researchers may perform follow-up lab investigations and whole-genome sequencing to examine a variety of basic and clinical microbiology and evolutionary theories using phages and/or bacteria collected during treatment. With our enhanced comprehension of the human microbiome and its interactions with human immunology, additional study into possible phage and immune system links in the treatment of infections is also necessary [56].

In order to attach and infect a bacterium, a phage may be chosen against the expression of the virulence factor. Selection against virulence factors may be more effective than usual because some of these factors, like capsules, have been demonstrated to mask antigenic sites [80], offer a certain level of antimicrobial resistance [81], and stop the macrophage from phagocytosing [80]. Bacterial attachment and invasion of epithelial cells can be inhibited by selecting phages against various virulence factors that can function as phage receptors, such as adhesins, pili, or secretion systems [82,83,84,85].

## 7. Significant Benefits of Phage Therapy over Traditional Antibiotics

Phage therapy offers several benefits compared to conventional antibiotics. Phage can kill both Gram-positive and Gram-negative bacteria [86,87]. Due to their ubiquity and abundance in every ecosystem, phages can be isolated quickly, which lowers their development costs compared to antibiotics. The phages can be isolated from a variety of settings, including soil, water, sewage wastewater, hospital wastewater, hot springs, feces, and the gastrointestinal systems of both humans and animals [88]. Phages are routinely sprayed on surfaces of preserved meats and cheeses to increase the shelf life of fridge-processed foods that are ready for consumption [89]. One of the most promising outcomes of phage treatment might be a decrease in C reactive protein (CRP) values, leukocyte counts, and erythrocyte sedimentation rates (ESR), as well as an impact on the inflammatory response to infection [90]. Phages have traditionally been described as having a high level of host specificity. However, new research has shown that phages can “jump” hosts, and the microbiota in the gut helps in this process [91]. Phage-host specificity could therefore change and adapt over time. The biggest benefit and largest drawback of phage therapy is related to its specificity.

When compared to antibiotic therapy, which disturbs the microbiome, phage therapy targets harmful bacteria directly. Phage therapy is free from adverse effects associated with microbiome disturbances, such as mucosal candidiasis, antibiotic-associated diarrhea, pseudomembranous colitis brought on by *Clostridium difficile*, and even long-term metabolic and immunological disorders. This is because it has no off-target effects [92]. On the other hand, phage specificity requires proper detection of the infections and causative agent identification, sometimes up to the level of the strain, a process that can be challenging and time- and resource-consuming [93].

Phage concentration rises at the infection location because it replicates inside the bacterial cells. Therefore, the existence and durability of phages prevent any possible development of secondary infection, which reduces the requirement for repeated doses to treat infectious disorders and ultimately improves the effectiveness of therapy and they can treat infections that would resist antibiotic therapy. Additionally, because phages spread quickly throughout the body, they can reach organs (like the prostate gland, bones, and the brain) that are difficult for drugs to reach [94].

Phages are thought to be a successful remedy against MDR, XDR, and PDR bacteria because they lack cross-resistance to antibiotics and mechanisms established by bacteria to resist medications that prevent interfering with phage efficacy [52,95,96]. Despite developing resistance to a particular phage, bacteria can still be infected by phages that target different cell surface receptors, such as those that target proteins, teichoic acid, and lipopolysaccharides [94]. When multiple types of phages are infecting the same species and strains, using a cocktail of phages has some benefits, including a greater effect on the targeted bacteria and a lower risk of the generation of phage-resistant bacteria [97].

## 8. Clinical Suspicion of IE

The clinical presentation of IE varies significantly and can take the shape of an acute, subacute, or chronic disease depending on the underlying heart abnormalities [bicuspid aortic valve, mitral valve prolapse, rheumatic valve disease, congenital heart disease, prior IE, patients with implanted cardiac devices (permanent pacemakers/implantable cardioverter-defibrillator) and prosthetic heart valves], pre-existing comorbidities [intravenous drug administration, chronic kidney disease (particularly dialysis patients), chronic liver disease, cancer, old age, corticosteroid usage, poorly managed diabetes, an indwelling venous access line, and an immunosuppressed state (along with HIV infection)], and the causative microorganisms. Up to 90% of patients suffer from fevers, night sweats, exhaustion, and a lack of appetite; 25% of patients suffer from signs of embolic phenomena [11]. For those who have predisposing risk factors such as heart murmurs, vasculitis, and embolic manifestations linked with IE a diagnosis of IE should be carefully examined [11]. Three sets of blood cultures should typically be performed before beginning antimicrobial medication; this will successfully detect bacteremia in up to 98% of patients [13,98]. Contrarily, the most common cause of culture-negative endocarditis is prior administration of antibiotic therapy, which leads to untargeted antibiotic treatment, unreliable diagnostics, and typically, lengthier and more toxic treatment regimens [11].

## 9. Microbiological Diagnosis

Positive blood cultures are essential for determining a diagnosis of IE and offer organisms for identification and susceptibility testing. Persistent bacteremia in numerous culture bottles of a typical organism is extremely significant. After consulting with an infection specialist, prolonging the time that blood culture bottles are incubated and performing serological testing should be carried out if blood cultures show no growth and the clinical suspicion of infection with IE is still high, particularly if there has not been any prior exposure to antibiotics. It is important to take into account possible causes of culture-negative endocarditis, including *Coxiella burnetii*, *Bartonella* species, *Tropheryma whipplei*, and various fungi, including *Aspergillus* species. In patients undergoing valve surgery for endocarditis, the infecting organism is often detected using a polymerase chain reaction (PCR) analysis of the valve tissue. If all microbiological testing is negative, sufficient investigation and testing should be carried out to exclude hypercoagulable diseases, systemic lupus erythematosus (Liebman–Sacks endocarditis), trauma, and non-bacterial thrombotic (marantic) endocarditis linked to malignancy [11].

## 10. Management and Treatment of IE

“The endocarditis team”, devoted personnel located in a reference center, should oversee the management of IE. Infectious disease experts and/or microbiologists, cardiologists with a focus on valvular heart disease or cardiac imaging, cardiac surgeons, and experts in cardiac devices should be included in this team. Since up to 30% of patients will develop symptomatic neurological episodes, access to specialists in neurology, neurosurgery, and congenital heart disease is necessary. This IE team strategy permits early, guideline-directed referral to surgery, suitable antibiotic prescription regimens, accessibility to modern imaging, close monitoring for complications, and follow-up after treatment is finished. One-year mortality is predicted to be roughly decreased by half in this environment [13].

Simple IE can typically be handled locally with regular communication with the reference center’s IE team. A specialized IE team should address complicated IE with heart failure, significant valve incompetence, structural damage (abscess, perforation, or fistula formation), and embolic or neurological sequelae. All IE cases should be regularly discussed at the reference center to decide on the best course of antibiotic therapy, how long it should last when surgery is necessary, and what kind of follow-up is necessary [11].

### 10.1. Antibiotic Therapy

IE was always lethal before antibiotics were developed. To successfully treat this illness, the right bactericidal regimen must be chosen and provided for the right amount of time; suggested published protocols for common organisms’ recommendations differ only slightly from one another. Since there is little clinical evidence to support gentamicin use, it has been removed from the majority of treatment recommendations for methicillin-sensitive *S. aureus* (MSSA). Amoxicillin and ceftriaxone are therefore suggested in the European guidelines and are particularly helpful in individuals with renal impairment. There is also growing experience with utilizing ceftriaxone as a synergistic treatment in enterococcal endocarditis [13].

The type of valve implicated, and the resistance pattern of the organism, determine the length of treatment and which antibiotics are used. The quickest recommended course of treatment for native valve endocarditis caused by the penicillin-susceptible *viridians* group or *S. gallolyticus* is a Ceftriaxone 2 gm IV combined with gentamicin 3 mg/kg IV every 24 h administered for two weeks [99]. Another viable regimen is aqueous penicillin G 12 to 18 million units every 24 h through continuous IV drip or in four to six equally divided doses. Ceftriaxone 2 gm IV/24 h for 4 weeks is an alternative regimen. When prosthetic valves are involved, these infections often require a minimum of a 6-week therapy with 24 million units of penicillin G or 2 g of ceftriaxone with or without 3 mg/kg of gentamicin/24 h [100].

Individuals who are susceptible to staphylococcal infection often need longer-term antibiotic treatment. MSSA infections of the native valve can be treated with 6-week courses of either cefazolin (2 gm every 8 h) or nafcillin (2 gm/4 h). A typical course of treatment for methicillin-resistant *S. aureus* (MRSA) infections includes daptomycin 8 mg/kg daily for six weeks or vancomycin 15 mg/kg/12 h. It should be noted that due to the lack of clinical benefit and concomitant renal damage, gentamicin dual therapy is no longer advised for MSSA or MRSA infections [99,101]. Staphylococcal infections of the prosthetic valve generally require identical treatment, although rifampin and gentamicin must be added. In addition to the nafcillin regimen mentioned above, patients with prosthetic valve MSSA illness should receive gentamicin 3 mg/kg IV in 2 to 3 divided doses along with rifampin 900 mg IV in two to three evenly divided doses every 24 h for 2 weeks and 6 weeks, respectively. MRSA cases should receive the same regimen of gentamicin and rifampin in addition to vancomycin [101,102].

Combination regimens, for instance, combining an aminoglycoside like gentamicin for 4–6 weeks with ampicillin or penicillin G, are necessary for treating both native and prosthetic valve enterococcal infections since beta-lactam monotherapy lacks bactericidal efficacy against *enterococci*. It is interesting that a dual beta-lactam regimen, such as ampicillin + ceftriaxone, has the proper level of bactericidal action against *Enterococcus faecalis* and can be used for that reason [103]. It should be noted that penicillin resistance calls for a combination of vancomycin and gentamicin therapy; however, emerging penicillin, gentamicin, and vancomycin resistance may call for linezolid or daptomycin therapy [100].

Generally, recommendations for antimicrobial treatment are always changing and should be examined regularly. Early infectious disease consultation is recommended to better direct and assist in developing appropriate antibiotic medication courses. Two blood cultures should be taken every 24 to 48 h as an extra concept of medical management to guarantee the elimination of bloodstream infection and to guide continuous antimicrobial medication [99].

Early surgical intervention is required in cases of acute heart failure, severe infection with local consequences, and recurrent arterial embolization, which may include valve repair instead of valve replacement. Surgery is frequently necessary within 24 h of the onset of heart failure symptoms brought on by acute valvular dysfunction. The AHA/ACC further recommends early surgical treatment before the completion of the first antibiotic course if there is a concomitant paravalvular abscess, atrioventricular block, or the existence of harmful infiltrative lesions [104].

Cardiothoracic surgery patients are more vulnerable to potentially lethal infections, and surgical site infections considerably increase the risk of postoperative death. Since bacteria usually form biofilms on implant surfaces that are highly resistant to antibiotics, implant-associated infections frequently turn into chronic illnesses. *S. aureus* is one of the most common bacteria connected to IE, and its prevalence has increased in recent years [105].

### 10.2. Phage Therapy

**Figure 2:**
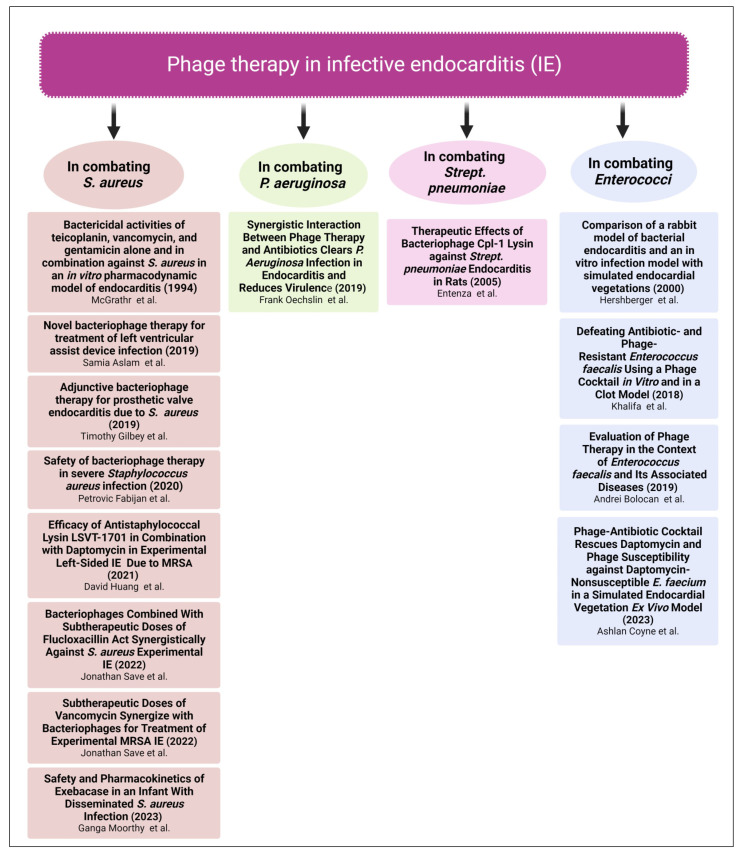
Published clinical studies regarding phage therapy in IE, including clinical trials and case studies [106,107,108,109,110,111,112,113,114,115,116,117,118,119]. Created with Created with BioRender.com.

Several clinical trials and case studies demonstrated and proved the promising effects of phage therapy in combating different resistant microorganisms causing IE as listed in Figure 2. 

#### 10.2.1. Phage Therapy in Combating *S. aureus*

**Figure 3:**
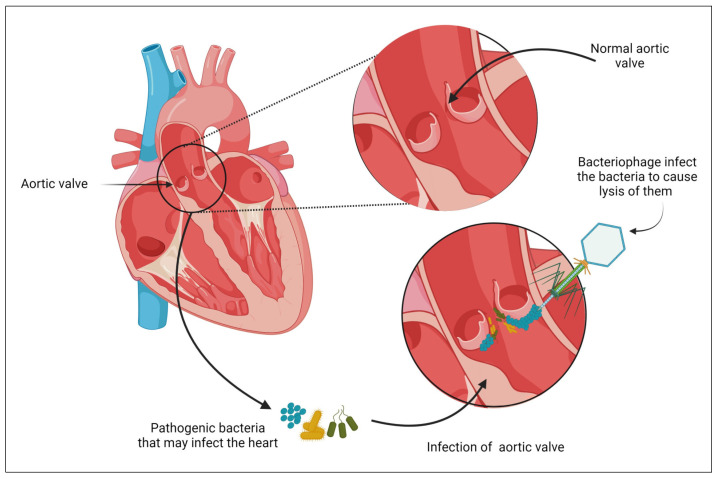
Phage therapy as a novel approach to combat IE. In the case of multidrug-resistant pathogens, phage therapy has been suggested as a salvage treatment for bacterial endocarditis. Created with BioRender.com.

As mentioned above, the most prevalent bacteria causing acute IE on both natural [120] and artificial valves [121] is *S. aureus*. Presently, the main treatment for *S. aureus* IE is a 4- to 6-week course of intravenous antibiotic therapy. If necessary, heart valve surgery is also an option [99]. Even most treatment strategies are linked to significant morbidity and mortality, with mortality rates in patients with prosthetic valve infection approaching 50% [122]. Novel approaches that might enhance outcomes for IE patients are still needed. Phage therapy is considered a virus used to treat bacterial infections and has been proposed as a salvage therapy (Figure 3), particularly in the context of MDR organisms [123].

Phage treatment has not yet received enough support from randomized controlled trials to be widely used; nevertheless, the currently available data suggest that phage therapy can complement or serve as a beneficial alternative to antibiotic therapy for the treatment of *S. aureus* infections, including burn and chronic wound infections [124,125], prosthetic joint infections [126,127], severe infections after cardiothoracic surgery [128], ventricular-assist device infections [129], and keratitis [130].

A three-phage cocktail has recently been tested for its safety and effectiveness in treating patients with *S. aureus* IE or *S. aureus* aortic graft infections, according to two Australian case studies [106,107]. It is encouraging to note that the addition of phages to antibiotic therapy increased the infection control and healing process. Nonetheless, there have been instances of treatment failure and/or recurrence, some of which have had deadly outcomes [108].

In a study by Save et al. [108], a mouse model of experimental endocarditis was used to test the effectiveness of a phage cocktail composed of the Herelleviridae phage vB SauH 2002 and the Podoviriae phage 66 against a MSSA strain both in vitro and in vivo. Six hours after the bacterial challenge, the animals received (1) the phage cocktail treatment, (2) a subtherapeutic dose of flucloxacillin, (3) a phage cocktail and flucloxacillin combination, or (4) saline. The main result was the number of bacteria in cardiac vegetation after 30 h. Phage burdens in the blood, spleen, liver, kidneys, and cardiac vegetation at 30 h were secondary outcomes [108]. Phages worked together in vitro to destroy *S. aureus* planktonic cells, and the cocktail worked with flucloxacillin to destroy biofilms. The phage cocktail produced a bacteriostatic effect in the infected animals. Low-dose flucloxacillin was added, which increased bacterial suppression. Notably, 30 h after receiving the combined medication, 9 out of 12 rats had sterile vegetations [108].

As we said previously, endocarditis caused by *S. aureus* is still associated with high rates of morbidity and mortality [131], particularly if brought on by MRSA [132]. Renal failure has been a clinical complication of vancomycin treatment [133] and the development of vancomycin-intermediate *S. aureus* (VISA) [134]. Thus, MRSA IE requires alternate and/or supplemental therapy. Thankfully, phage treatment produces ideal results in the management of this condition, as demonstrated by Save et al. [109].

Phages have primarily been studied in conjunction with antibiotics, which is a marked contrast to the majority of experimental settings [135]. Antibiotic use might be a double-edged sword if it lowers the population of bacteria necessary for phage replication [135]. Save et al. found a synergistic bactericidal activity of a new anti-*S. aureus* two-phage cocktail when combined with beta-lactam and flucloxacillin for the treatment of experimental MSSA IE. They also discovered that flucloxacillin drastically changed the phages’ in vivo pharmacokinetic (PK) profile [108]. Another study by Save et al. demonstrated the possibility of using the same phage cocktail in combination with the glycopeptide vancomycin to treat the experimental rat model of MRSA IE. Also, they assessed the impact of vancomycin on the PK profile of phages [109]. Vancomycin in combination with a 1:1 phage cocktail made up of *Herelleviridae* vB SauH 2002 and *Routreeviridae* 66 was tested for effectiveness using an experimental rat model of MRSA IE [109]. Animals were given one of two treatments six hours after being inoculated with approximately five log10 colony-forming units (CFU) of MRSA strain AW7: (i) saline, (ii) an equimolar two-phage cocktail, (iii) vancomycin (at a dose mimicking the kinetics in humans of 0.5 g b.i.d.), or (iv) a combination of both. The results were assessed for bacterial and phage loads in vegetation, as well as in the kidney, spleen, liver, and blood [109]. They found that the growth of strain AW7 in cardiac vegetation could not be stopped by a phage cocktail on its own. However, a statistically significant reduction in growth occurred when subtherapeutic dosages of vancomycin were added to the phage cocktail. It was discovered that the administration of vancomycin had a considerable effect on the local concentrations of phages in the studied organs and vegetation [109].

A novel, recombinantly generated, bacteriophage-encoded lysin called LSVT-1701 (formerly known as SAL200) targets *staphylococci* in particular by hydrolyzing their cell walls enzymatically. To test the combined effectiveness of the lysin LSVT-1701 and daptomycin, they used the rabbit model of infective endocarditis of the aortic valve. Methicillin-resistant *Staphylococcus aureus* (MRSA) levels in target tissue were significantly decreased when LSVT-1701 and daptomycin were combined. Both lysin dosage regimens sterilized all target tissues when administered for four daily doses along with daptomycin. These results imply that additional clinical evaluation of LSVT-1701 as an adjuvant therapy for the management of invasive MRSA infections is needed [110].

Aslam et al. employed phage therapy as an adjunct to antibiotics for the first time to treat infection of the left ventricular assist device. A 65-year-old male patient with nonischemic cardiomyopathy experienced *S. aureus* device infection, which required many hospital stays, surgical debridement, and long-term injectable antibiotics. This man was not eligible for a heart transplant due to his ongoing recurring infections. For 28 days, the antistaphylococcal phage cocktail AB-SA01 was given intravenously every 12 h, coupled with cefazolin every 8 h and minocycline twice daily. The patient’s condition got better, and his sternal cultures for MSSA were negative at the end of the first week and for the duration of the rest of the therapy. With this method, the wound’s granulation tissue was healthier and less purulent. This patient was able to have a transplant as a result, and seven months later, he was healthy and the illness had not returned [111].

Recently Gilbey et al. reported that the antistaphylococcal phage cocktail ABSA01 was successfully used for the first time intravenously to treat severe staphylococcal sepsis with prosthetic valve endocarditis [107]. They studied the effect of the ABSA01 phage cocktail on the patient who was unable to have heart valve surgery due to severe *S. aureus* IE on a mechanical aortic valve, a native mitral valve, and maybe a paravalvular root abscess [107]. Blood cultures consistently came back positive, even though the bacterial strain was antibiotic-susceptible, and the patient was being treated with a high dose of ciprofloxacin, rifampicin, and flucloxacillin. The patient’s health improved after receiving an intravenous ABSA01 phage cocktail injection twice daily for 14 days along with antibiotics; the patient became afebrile, the inflammatory markers decreased, the persistent bacteremia disappeared, and the patient made a full recovery [107].

In another study, Petrovic Fabijan et al. also studied the effect of the ABSA01 phage cocktail on 13 patients who had severe *S. aureus* infections as an additional therapy. The patients received ABSA01 intravenously twice daily for 14 days, while their clinical, hematological, and blood biochemical parameters were tracked for 90 days. The assessment of safety and tolerability was evaluated and presented by pain and redness at the infusion site and systemic adverse reactions, such as fever, hypotension, tachycardia, diarrhea, development of renal or hepatic dysfunction, and abdominal pain. Their data show that ABSA01 delivered in this manner is safe in cases of severe *S. aureus* infections, including IE and septic shock, as no adverse effects were documented. The effectiveness of ABSA01 will require further controlled experiments, although no in vivo phage resistance has been observed [106].

A possible adjunctive therapy for severe MRSA infection is exebacase, an antistaphylococcal lysin produced from a bacteriophage-encoded gene. This research only covers one patient, making it difficult to determine if clinical improvement was brought on by exebacase alone or by prolonged antibiotic therapy. Despite these drawbacks, the new agent exebacase has the potential to be an effective adjunctive treatment for children with severe MRSA infections [112].

#### 10.2.2. Phage Therapy in Combating *Streptococcus pneumoniae*

Cpl-1, a pneumococcal phage lytic enzyme, was investigated in rats with experimental endocarditis caused by *Streptococcus pneumoniae* WB4. A high-dose Cpl-1 regimen eradicated *pneumococci* from blood in 30 min and reduced bacterial titers in vegetations in 2 h. The rapid bacterial lysis caused by Cpl-1 treatment significantly boosted cytokine production [113].

#### 10.2.3. Phage Therapy in Combating *Pseudomonas aeruginosa*

Recently, a fairly uncommon bacteria that causes IE, *Pseudomonas aeruginosa* (*P. aeruginosa*), was the subject of an experimental investigation utilizing a phage cocktail alone or in combination with the antibiotic ciprofloxacin. However, the research showed that a single-dose phage reduced *P. aeruginosa* in the clots by 7 logs [114]. Phage and ciprofloxacin monotherapy each resulted in a 2.5-log reduction in the rat model, while the combination showed a strong synergistic impact with >6-log killing [114]. After 24 h, phage-resistant mutants were seen in vitro but not in vivo; however, this could be avoided by mixing the phages with ciprofloxacin [114]. In fact, due to the selection of PRVs (phage-resistant variants) having acquired mutations in either the *galU* gene, which codes for LPS synthesis or the PilT ATPase, which is involved in pilus retraction, bacterial regrowth caused by phage resistance could be demonstrated after 24 h in vitro. In vivo, PRVs were not seen before or after phage therapy treatment, which, depending on the mode of phage administration, reduced the bacterial load by 2.3–3 log colony-forming units (CFU). Lastly, ayu fish that had been orally infected with *P. plecoglossicida* were given phage therapy, and the appearance of PRVs with reduced virulence that had not been seen in vivo was also verified [136]. When administered intramuscularly to fish, PRVs chosen in vitro were less virulent than before [136].

#### 10.2.4. Phage Therapy in Combating *Enterococci*

*Enterococci* now account for about 10.5% of all instances of IE [6]. The gastrointestinal tract (GIT) typically harbors the Gram-positive non-spore-forming bacterium genus *Enterococci*, which now has 35 identified species [137], including *E. faecalis*. MDR strains of *Enterococci* have become more prevalent due to the remarkable capacity of these bacteria to adapt to a variety of circumstances and their tendency to develop antibiotic resistance [137]. *E. faecalis* is primarily known as a normal flora of the human gut, but it can also function as an opportunistic pathogen that can breach the mucosal barrier to cause systemic infections [138,139]. *E. faecalis* and *E. faecium* make up more than 90% of the bacterial isolates frequently found in clinical collections (blood and other samples from infectious sites) [140,141]. Bacteremia, meningitis, IE, and urinary tract infection in addition to root canal infections are among the life-threatening illnesses commonly associated with *E. faecalis* [115].

Furthermore, *E. faecalis* is inherently resistant to a wide range of antibiotics, including penicillin, piperacillin, ampicillin, vancomycin, and imipenem, all of which have bacteriostatic rather than bactericidal effects [142]. Vancomycin-resistant *E. faecalis* (VREF) and other vancomycin-resistant *enterococci* (VRE) have caused significant worries during the last 10 years. In the context of a combined fatality rate of 20–40% for IE caused by *E. faecalis* and *E. faecium*, *E. faecalis* accounts for about 97% of cases [143].

These infections are thought to be challenging to treat because of the ineffective activity of β-lactams and the capacity of *E. faecalis* to produce biofilms. Therapy for severe infections caused by *E. faecalis* frequently involves combinations of antibiotic medications. Even these antibiotic treatment choices are restricted since 50% of isolates have significant levels of aminoglycoside resistance, which is produced by enzymes that alter aminoglycosides. These enzymes prevent the synergistic bactericidal effect that results from mixing an aminoglycoside with a cell wall-active agent [143,144].

The effectiveness of single phages or phage cocktails in the management of bacterial biofilms is described in numerous trials. For instance, pathogenic bacteria create biofilms like *Streptococcus mutants* [145], *S. aureus* [146], *E. coli* [147], *E. faecalis* [148], and *P. aeruginosa* [149], phages can disrupt that biofilm [115]. Compared to traditional antibiotics, phage therapy is more effective against biofilms because the phages infect the bacteria from the top layer. Upon the reproduction cycle, they generate a new virion progeny that affects the bottom layer(s). This layer-by-layer method of operation successfully eliminates biofilms [148,150]. The most popular technique for researching biofilm development and evaluating the efficacy of antimicrobial agents is microtiter plate analysis. The viewing of biofilm matrices both before and after phage therapy can also be accomplished with the use of more sophisticated techniques, such as confocal microscopy [151]. This technique has been used to demonstrate how well phage EFDG1 reduced *E. faecalis* V583 biofilms that were two weeks old [152]. The static biofilm of *E. faecalis* strains JH2-2 and V583 that had developed on coverslips was diminished by the genetically modified phage phiEf11. A 10–100-fold reduction in viable cells (CFU/biofilm) was seen after 24 and 48 h of incubation [115].

As thrombin transforms fibrinogen into fibrin, a structural protein that assembles into a polymer, clots are gel-like collections of blood [153]. The effectiveness of antibiotics in the treatment of IE has been effectively tested using an in vitro fibrin clot model [116], proving that the bacterial strains *E. faecalis* [154], *E. faecium* [116,155] *S. aureus* [117], and *Bacillus cereus* [155] are capable of causing clotting in vitro. The effectiveness of individual phages and phage mixtures has recently been demonstrated using the in vitro fibrin clot model [118]. Vancomycin-resistant and sensitive *E. faecalis* strains were seeded into plasma by the authors, who then used CaCl2 and bovine thrombin to cause plasma coagulation. The resulting clots received phage treatment at a concentration of 108 PFU/mL. The phage(s) EFDG1 and EFLK1 significantly reduced bacterial numbers by 3–6 logs following treatment [115].

*Enterococcus faecium* is challenging to treat because it is increasingly resistant to the majority of clinically useful drugs. The gold standard of treatment is daptomycin (DAP), although even large dosages of DAP (12 mg/kg body weight/day) were unable to completely eliminate some vancomycin-resistant bacteria. A DAP-ceftaroline (CPT) combination may increase β-lactam affinity for target penicillin-binding proteins (PBP), but DAP-CPT did not show therapeutic efficacy against a DAP-nonsusceptible (DNS) vancomycin-resistant *E. faecium* isolate in a simulated endocardial vegetation (SEV) pharmacokinetic/pharmacodynamic (PK/PD) model. It has been suggested to use phage-antibiotic combinations (PAC) to treat resistant, high-inoculum infections. They tried to find the PAC with the greatest bactericidal action as well as phage and antibiotic resistance prevention/reversal in a SEV PK/PD model against DNS isolate R497. Modified checker-board MIC and 24 h time-kill analyses (TKA) were used to analyze phage-antibiotic synergy (PAS). Then, 96 h SEV PK/PD models against R497 were used to evaluate human-simulated antibiotic dosages of DAP and CPT with phages NV-497 and NV-503-01. The PAC of DAP-CPT mixed with phage cocktail NV-497-NV-503-01 showed synergistic and bactericidal activity, displaying a significant reduction in viability down to 3-log10 CFU/g. Additionally, this combination showed isolate DAP resensitization. Evaluation of phage resistance following SEV revealed that PACs containing DAP-CPT prevented phage resistance. The study’s findings offer novel information about the bactericidal and synergistic effectiveness of PAC against a DNS *E. faecium* strain in a high-inoculum ex vivo SEV PK/PD model, as well as the following DAP resensitization and phage resistance prevention [119].

## 11. Conclusions

Phage therapy alone or a combination of phages with antibiotics in the treatment of various bacterial infections such as IE has promising results and successful trials, but more and more efforts are needed to evaluate the efficacy and safety of phage therapy.

## Data Availability

Not applicable.
